# In this month’s Bulletin

**DOI:** 10.2471/BLT.24.000624

**Published:** 2024-06-01

In the editorial section this month, Mahmut Uludağ and colleagues (374) present a database that tracks genetic mutations in SARS-CoV-2. Jenny JW Liu et al. (375) discuss ways to improve workplace support for health workers. 

Gary Humphreys (378–379) reports on vaccine development targeted to antimicrobial resistant pathogens. Ivanilda (Vanny) Reis talks to Gary Humphreys (380–381) about how physical activity throughout the life course improves health and well-being.

## Lebanon

### Overuse of antibiotics in pregnant refugees

Christine Al Kady et al. (389–399) improve treatment patterns for urinary tract infections. 

## Uganda 

### Managing cervical cancer and HIV

Julius Namonyo Kalamya et al. (382–388) evaluate service provision.

### Tuberculosis screening, testing and care 

Stavia Turyahabwe et al. (400–409) assess the effectiveness of a community-based intervention.

## United Kingdom of Great Britain and Northern Ireland 

### Measures to reduce sugar consumption

Kawther M Hashem et al. (432–439) study policy outcomes.

## Global

### Floods as a public health issue

Qiao Liu et al. (410–420) present data on global impact from 1990–2022.

### Health literacy and tuberculosis outcomes

Arohi Chauhan et al. (421–431) review the evidence for an association.

### Better pandemic preparedness 

Thomas J Bollyky and Michael Bang Petersen (440–447) summarize ways to improve public trust and compliance with pandemic responses.

### How much harm does alcohol cause to non-drinkers? 

Carolin Kilian et al. (448–452) propose a research and policy agenda.

### Analysis of human, environmental and animal health risks

Monika Ehling-Schulz (453–456) introduce a risk negotiation framework.

